# Economic burden of stroke in a large county in Sweden

**DOI:** 10.1186/1472-6963-12-341

**Published:** 2012-09-26

**Authors:** Josefine Persson, José Ferraz-Nunes, Ingvar Karlberg

**Affiliations:** 1 Sahlgrenska Academy, Gothenburg University, Gothenburg, Sweden; 2 University West, Trollhättan, Sweden

**Keywords:** Burden, Cost of illness, Health economics, Incidence cost, Stroke, Sweden

## Abstract

**Background:**

Stroke remains to be a major burden of disease, often causing death or physical impairment or disability. This paper estimates the economic burden of stroke in a large county of 1.5 million inhabitants in western Sweden.

**Methods:**

The economic burden of stroke was estimated from a societal perspective with an incidence approach. Data were collected from clinical registries and 3,074 patients were included. In the cost calculations, both direct and indirect costs were estimated and were based on costs for 12 months after a first-ever stroke.

**Results:**

The total excess costs in the first 12 months after the first-ever stroke for a population of 1.5 million was 629 million SEK (€69 million). Men consumed more acute care in hospitals, whereas women consumed more rehabilitation and long-term care provided by the municipalities. Younger patients brought a significantly higher burden on society compared with older patients due to the loss of productivity and the increased use of resources in health care.

**Conclusions:**

The results of this cost-of-illness study were based on an improved calculation process in a number of fields and are consistent with previous studies. In essence, 50% of costs for stroke care fall on acute care hospital, 40% on rehabilitation and long-time care and informal care and productivity loss explains 10% of total cost for the stroke disease. The result of this study can be used for further development of the methods for economic analyses as well as for analysis of improvements and investments in health care.

## Background

Stroke continues to be a major burden of disease [1,2] even when the risk of premature death has been reduced [3]. Several assessments of the direct and indirect costs have been published [4-9]. In Sweden, the comprehensive statistics from providers on the utilization of in-hospital, municipal and long-time care make it possible to evaluate the costs from a wide societal perspective in detail [10]. Sweden, like many Organisation for Economic Co-operation and Development (OECD) countries, faces a problem of an increasing fraction of elderly persons, who will in the long run lead to a heavy burden on society and the working population [11]. Thus, the financial burden of diseases such as stroke is important.

**Figure 1:**
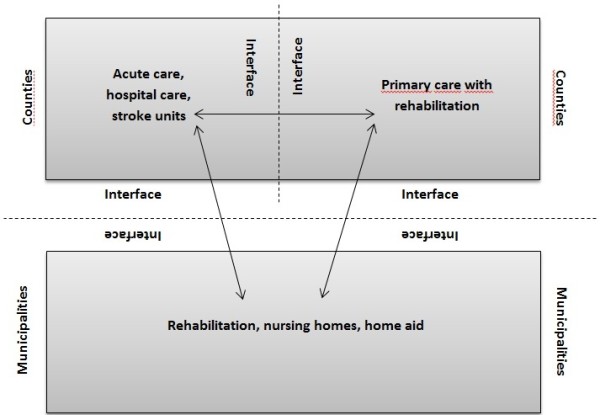
**Providers in Swedish health care system.** Source: Integrated Care in Europe. Description and comparison of integrated care in six EU countries. Elsevier Gezondheidszorg, 2003.

The Swedish health care model is based on the goal to provide good health and high-quality care on equal terms for the entire population. Residents in Sweden have equal rights of access to care based on the medical needs and in accordance with evidence-based medicine and proven experience. Those who are in greater need should take precedence, regardless of their ability to pay, place of residence, social status or other factors that are medically irrelevant. Health care should also be cost-effective, and the resources within the health care system should be used so that maximum gain is reached to meet the needs of the population. The Swedish health care system is highly decentralised with three levels, the state, the counties and the municipalities. The state sets the regulations but does not provide care. The counties provide all health care including hospital, acute care and primary care whereas the municipalities provide rehabilitation, long-time care and home aid (Figure 1). All units of care regardless of the provider are financed by local taxes.

A health economic analysis of a disease implies that there is information available regarding the use of resources at different levels in the chain of health care and society. By using the social security number, Sweden has a unique ability to follow one patient throughout the whole chain of health care within the Swedish administrative registries. In this paper, relevant data were selected to provide a complete picture of the burden of stroke in Västra Götaland, a county in western Sweden including health care consumption, municipal care, potential productivity loss and informal care by relatives.

The aim of this exploratory study was to present the societal costs of first-ever stroke during 2008 in Västra Götaland, a large county in western Sweden, which has 1.5 million inhabitants. The study identified, quantified and valued all costs for health care, rehabilitation, long-term care and home aid and potential productivity loss. Also, all costs for patients and families during the first 12 months after the first-ever stroke as well as lifetime costs, were estimated. This study analysed the costs arising due to the increase in resource consumption due to stroke.

## Methods

### Epidemiology

Stroke is a clinical syndrome with several different pathologies [12]. In this study we defined stroke as International Classification of Disease, 10^th^ revision (ICD-10) codes I61 (intracerebral haemorrhage), I63 (cerebral infarction) and I64 (stroke, not specified as haemorrhage or infarction) [13]. According to local data of health care consumption from the county council, 4,242 stroke events were reported in 2008, of which 3,074 cases (72%) were first-ever stroke. This proportion is in accordance with a previous study conducted in Sweden [5]. The sex distribution in the whole group was equal (Table 1). However, in the age group below 55, the proportion of men was 67%, whereas in the age group above 85 the proportion of women was 66%. This could partly be explained by women living longer than men [14]. Comorbidity such as atrial fibrillation and hypertension indicates risk factors for first-ever stroke (Table 1). Health-related quality of life decreased with age, i.e. patients over 85 had the lowest self-reported health-related quality of life. Women had lower self-reported health-related quality of life and impaired mobility compared with men (Table 2).

**Table 1:** Demographic and clinical variables for patients with first-ever stroke in western Sweden during 2008

**Patient characteristics**	
Sex, distribution	%
Female	49.2
Male	50.8
Age, distribution	
<54 years	6.2
55**–**64 years	13.1
65–74 years	22.0
75**–**84 years	32.9
>85 years	25.8
Comorbidity	
Atrial fibrillation	27.3 (5*)
Diabetes mellitus	18.9 (5*)
Hypertension	55.8 (19*)
Previous TIA	6.3 (0.2*)
Smoker	13.7 (13*)
ICD-10, distribution	
I61	11.2
I63	84.9
I64	3.9

Total number of patients n = 3,074.

Data source: Local administrative register and The Stroke Register.

*Reference value for the age-matched Swedish population, according to The Swedish National Institute of Public Health.

**Table 2:** Age-related differences in health-related quality of life and mobility three month after first-ever stroke onset for patients in western Sweden during 2008

**Age, sex**	**EQ-5D**	**Impaired mobility,%**
<54 years	68.3 (75.0*)	13.5
55-64 years	70.7 (73.0*)	10.9
65-74 years	68.4 (72.4*)	16.9
75-84 years	64.9 (68.8*)	31.9
>85 years	58.5 (64.6*)	48.5
Female	64.1 (73.5*)	33.0
Male	70.3 (73.8*)	19.7

Total number of patients n = 3,074.

Data source: The Stroke register.

*Reference value for the Swedish population. Source: Ferraz-Nunes J: Hälsa, ett värde utan pris (Health, a value without price), Holmberg & Weibull (red). Det nya samhället (The new society), SOM. Sweden: Gothenburg University; 2000:93–110.

### Data source

**Figure 2:**
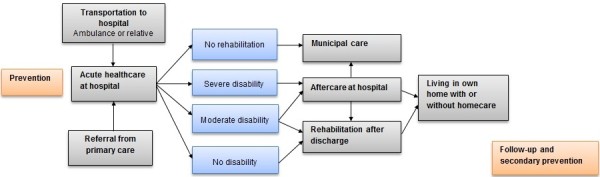
Chain of health care for stroke survivors in Sweden.

To identify, quantify and value events and contacts attributable to stroke, identifying the chain of healthcare is important. It is then possible to estimate all costs occurring in each step of the chain (Figure 2). Retrospective data were extracted from the local administrative register and a national clinical register, The Stroke Register, to calculate resources attributable to stroke. The clinical register contains information on stroke admissions in Sweden [15], comorbidity, health-related quality of life, medication, admissions to municipal care and dependence on relatives. Collection of data into the national clinical registries is based on the law SFS 2008:355. Local data of health care consumption were extracted from the county council of Västra Götaland [10], containing health care consumption for inpatients care as well as outpatient care. Data for inpatient care contained dates of admission and discharges, primary diagnosis (ICD-10) and type of ward, and data for outpatient care contained date visit, primary diagnosis (ICD-10), type of medical personnel and type of clinic/facility. To calculate the excess cost for stroke, health care consumption data were extracted one year before and one year after the stroke onset for each individual. Collection and utilisation of data for assessment is based on the law SFS 2008:355 and the Health Act HSL 36 §. To complement and verify the local administrative data, macro data were extracted from The Stroke Register. We did not use individual data from this source. We did not collect primary data and use individual data, and approval from the ethics committee was not relevant.

### Direct costs in health care

Data were collected, based in an incidence method, to estimate disease cost for first-ever stroke patients for a single year. Inpatient care costs were calculated according to age intervals, sex and diagnosis. The cost of emergency and inpatient care consists of the costs of all care episodes that began in the year 2008 for a period of 12 months after the stroke. Most patients had only one treatment episode, but some patients had more episodes during the 12-month period. Each episode includes basic, intensive care, nursing, diagnosis, blood transfusion and rehabilitation costs (Table 3). Costs for outpatient care varied to a larger extent than costs for inpatient care due to the differences between patients in age and disabilities caused by stroke. Cost-driving contacts after discharge were visits with doctors and nurses, rehabilitation with physiotherapists, occupational therapists, speech therapists and counsellors (Table 3). Data collection was based on number of visits over the 12-month period. Costs per contact and per hospitalisation day were based on prices in the county and includes overhead, equipment and personnel costs. The average cost per patient in each age group was calculated.

**Table 3:** Main unit cost per resource use items in hospital care, municipal rehabilitation and aid service, average income and informal care for patients with first-ever stroke in western Sweden during 2008

**Cost item**	**Unit**	**SEK**	**(€)**
Pre-hospital			
Ambulance transportation		2000	(220)
Own transportation		1000	(110)
Inpatient care	Per diem	5400	(600)
Outpatient care			
Medical specialist	Per visit	4000	(440)
General practitioner	Per visit	2000	(220)
Speech therapist	Per treatment	800–6100	(90–700)
Other	Per visit	1800	(200)
Municipal care			
Municipal home care	Per diem	480	(50)
Municipal assistant living	Per diem	1600	(180)
Average income	Per month	35000	(3900)
Informal care			
Working age	Per hour	200	(20)
Pension age	Per hour	70	(8)

Prices of year 2008 in SEK and (€).

Based on converting currency rate 1 June 2012 (1 SEK = €0,11).

Data source: see methods.

### Municipal cost

As this is a study of excess costs, municipal costs were calculated based on the change in the use of rehabilitation as well as long-term care and home aid before and after the stroke, respectively based on information from The Stroke Register. Municipal excess costs were small for individuals who had already used a great deal of care before the stroke. Costs for patients who died during the first three months after the stroke were limited. The average *per diem* cost was based on information from municipal professional workers.

### Informal care volume

Estimates of the volume of hours spent on informal care were based on the information in The Stroke Register, which was verified by interviews with caregivers and employees within the municipalities. According to the register, 2,076 patients reported that they were entirely or partly dependent on their informal caregiver. It was estimated that those who were entirely dependent on their informal caregiver received four hours each day and those who were partly dependent on their informal caregiver received four hours each week. For patients above 85, this volume of informal care was halved because data from The Stroke Register on this age group indicated consumption of municipal care to a larger extent before the stroke.

### Informal care costs

The opportunity cost method was used to calculate the socioeconomic value of informal care, meaning the value of the informal caregiver’s best alternative use for this time, which may be the loss of income including social security contributions and/or loss of leisure time. According to previous studies, informal care is mainly given by the patient’s partner [16-18]. Patients in the age group below 65 were estimated to have partners in working age, and were therefore estimated to have a loss of income of 200 SEK per hour based on the average income in western Sweden during 2008 calculated by from Statistics Sweden [19]. This includes an estimation of 25% on sick leave or disability pension. Patients above 65 were estimated to have partners not in working age and therefore to have a loss of leisure time of 70 SEK per hour, which is 35% [20] of the production loss value (Table 3).

### Cost for loss of productivity

Estimation of cost for loss of productivity was based on sick leave and early retirement due to the ICD-10 codes I61, I63 and I64 in the year 2008. This information was obtained from the social insurance authority, which registers all absence to work in Sweden. Another two weeks were added to the time the sick leave because this period is not included in the initial phase of sick leave covered by the insurance. The period was calculated in working days and then multiplied by average day-income including social security contributions based on the average monthly market income in the county [19] (Table 3). Total potential productivity loss was recalculated with a factor that takes into account absence from work due other causes such as unemployment and other sicknesses. By this, the employment status of the patients before and after stroke was taken in consideration and a measure of lost social potential production was calculated. The period was calculated in working days and then multiplied by average income including social security contributions, which is a measure of output from a market perspective. The potential productivity loss was estimated based on the human capital approach.

### Lifetime costs

Estimation of lifetime costs was based on estimated excess total cost in the first three years after first-ever stroke. The calculation of the first year corresponds to the follow-up of patients at the individual level indicating the exact real consumption of health care services, community care and production loss. In the second year, costs were estimated based on information about the discharge of patients. This information includes mortality, survival rates, degree of handicap, change in the need of community service, health care and long sick leave. Future development of costs after three years were adjusted based on data from clinical registries and other studies [5,21]. Future costs were discounted to present value using a discount rate of 3%. The structure of costs is substantially different in the first year after stroke compared with future costs. The weight of community costs increased after the first year.

## Results

### Excess total costs

This study estimates the total excess care costs in the first 12 months after first-ever stroke to be 629 million SEK (€69 million) per year for a population of 1.5 million in the Västra Götaland county, western Sweden (Table 4). The excess cost means that the consumption of non-stroke-related health care is ignored. Medical expenses relating to resources used in inpatient and outpatient care were estimated to be 49% of the total costs. Municipal care costs were estimated to be 34% of the total costs. Patient’s loss of productivity is equal to the value of lost production in society due to absence from work and was estimated to be 9% of the total costs. The socioeconomic value of the informal care costs for the first year after the stroke was estimated to be 6% of total costs.

**Table 4:** Excess total costs for all patients, excess cost per patient and lifetime costs for patients with first-ever stroke in western Sweden during 2008

**Cost item**			**Cost per 1,000 inhabitants**	
	**SEK**	**€**	**SEK**	**€**
Costs all patients				
Inpatient care costs	280000	(31000)	190	(21)
Outpatient care costs	22000	(2500)	15	(1.7)
Municipal care costs	230000	(25300)	150	(16.5)
Informal care costs total	39000	(4300)	25	(2.8)
Productivity loss total	58000	(6300)	40	(4.4)
Total costs	629000	(69000)	420	(46.2)
Cost per patient				
Average all patients	193	(21.2)		
Patients under 55 years	280	(30.8)		
Patients between 65–74 years	136	(15)		
ICD-10 code I61	202	(22.2)		
ICD-10 code I63	182	(20)		
ICD-10 code I64	161	(17.7)		
Lifetime costs				
All patients	768	(84.5)		
Patients under 55 years	2447	(269)		
Patients over 85 years	423	(46.5)		

Total number of patients n = 3,074. Prices of year 2008 in thousand SEK and (€).

Based on converting currency rate 1 June 2012 (1 SEK = €0,11).

### Excess cost per individual

The average excess cost per individual in the first year after a first-ever stroke was calculated to be 193,000 SEK (€21,200) (Table 4). However, this excess cost varied greatly between individuals depending on age, sex, and severity of the stroke. The average cost per individual below 55 was more than twice as high as the cost for individuals between 65 and 74. Of total costs, approximately 50% were related to health care and approximately 40% were related to municipal care. However, this ratio differs significantly between age groups. For individuals below 65, the proportion of health care costs was greater whereas for the older individuals the proportion of municipal care was greater. There were some differences when the costs per individual were grouped in the ICD-10 codes because a stroke caused by a cerebral haemorrhage (I61) incurs the largest cost per individual. Age-related differences in costs were similar among men and women; however, men used more resources in health care and women more municipal care (Table 5).

**Table 5:** Differences in excess costs per patient with first-ever stroke in western Sweden during 2008 for females and males

**Cost item**	**Female**		**Male**	
	**SEK**	**€**	**SEK**	**€**
Inpatient care costs	88	(9.6)	95	(10.5)
Outpatient care costs	6	(0.7)	5	(0.5)
Total healthcare cost	94	(10.3)	103	(11)
Municipal care costs	78	(8.5)	72	(8)
Total direct costs	172	(19)	175	(19)
Productivity loss total	17	(2)	21	(2.3)
Total costs	189	(21)	196	(21.5)

Total number of patients n = 3,074. Prices of year 2008 in thousand SEK and (€).

Based on converting currency rate 1 June 2012 (1 SEK = €0,11).

### Excess costs the first three years

**Figure 3:**
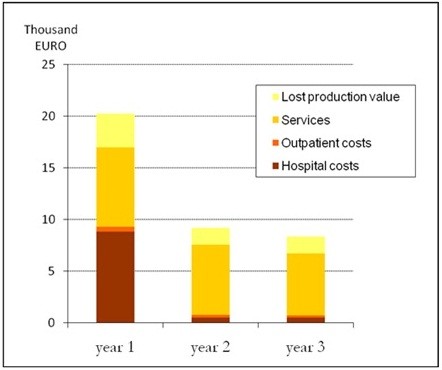
**Cost per patient during three years after first-ever stroke in western Sweden during 2008.** Year 2008 prices in thousand EURO.

Costs after the first year after a first-ever stroke were significant but had another structure compared with the first year. During the first year, health care cost accounted for the largest share of the cost whereas the proportion of municipal service costs exceeded health care costs after the first year (Figure 3). Lifetime costs are extended through the remaining years of life and were estimated to be 768,000 SEK (€84,500) in present value (3% discount) per individual. However, the lifetime cost per individual varied greatly depending on age. For patients below 55, lifetime costs were almost six times higher than for patients above 84. This variation was mainly due to productivity loss for the working population and in addition the health care and municipal care for rehabilitation and aid that is needed for several years.

### Generalizability and comparability

Costs for life time care are based on statistics for stroke and demography. Changing case-mix may have an influence on the assumptions. As indicated from our results, rising ages for patients with first stroke ever will lead to decreasing overall societal costs. Also, as a previous study [22] indicates, the incidence of stroke increases among the younger population, especially among younger women. These are probable scenarios and might affect the burden of stroke in a different way than this study indicates.

## Discussion

### Methodological considerations

The estimated excess cost in this study gives a value of the societal burden that could be saved if one stroke-incidence could be avoided. One of the main strengths of this study is the detailed information covering every part throughout the whole chain of health care in addition to societal costs. This study gives indication of the costs in the year 2008; however, there may be variations due to improvements the in health of the population as an effect of new treatments as well as preventions. By this, the result in this study can be used as a basis to evaluate new improvements. Also, this study could be used to initiate a discussion of cost and priorities. However, the calculations have some limitations. There is a lack of data at the individual level in municipal care because only the county health care registers data on individual level. Municipal care is for legal reasons not registered for single individuals, which makes it impossible to perform a regression analysis exploring causality. However, the calculation of average cost is not affected by this. Other published studies in the field had the same problem [23]. Also, there are some uncertainties in calculating the informal care due lack of systematic evidence in the area, which may have a significant influence in health economic analyses [11]. Thus, systematic data on informal care is an essential area for further research.

### Analysis of result

This study shows that the expenses for specialist health care in the first year causes most of the excess costs of stroke, but this cost varies between age groups. For individuals older than 75, most of the excess costs are within the municipal care, e.g. rehabilitation and long-time care. Within the health care, inpatient care costs dominated. The study also shows that the excess costs vary across age group, sex and diagnosis. Excess cost in the younger age group is significantly higher than in the older group due to more resources used in health and municipal care as well as productivity loss. Men consumed more resources within health care and had a higher level of productivity loss. Individuals who suffered a cerebral haemorrhage (I61) consumed more resources than other patients in all cost categories. Even though this kind of stroke affects only few individuals, the individuals who are affected are younger and require most resources, both within health care and municipal care. The estimated opportunity cost for informal care is an understatement due to lack of systematic data. However, this gives an indication of the family’s burden in monetary terms.

### Comparison with other studies

When comparing the result of this study with other studies on the topic, differences in calculation methods have to be considered. However, compared with previous studies in Sweden, our study showed similar results to other studies on an aggregate level. The most recent study [5] in Sweden shows that the lifetime-cost per individual was 725,000 SEK (€80,000) converted to 2008 year prices and with a discount rate of 3%. This figure represents an average for Sweden. The life-time cost in this study is calculated to be 768,000 SEK (€84,500).

Comparisons with studies in other countries add even more uncertainties due to different health care systems. A European study by Porsdal and Boysen [7] showed differences in the use of specific recourses within the health care chain. For example in Denmark and Finland, a large amount of rehabilitation resources are used. In our study, we only estimated the rehabilitation use to 10% compared with the 23% in Porsdal’s study [7]. In this study, the length of stay was about 12 days, whereas the corresponding figure in Porsdal’s study was 33 days [7]. This is an example of the different choices of resource allocation within the health care chain, which have an effect on the overall costs.

## Conclusions

The results of this cost of illness study are consistent with previous studies, although we have the cost calculation process in a number of fields. In essence, 50% of costs for stroke care fall on acute care hospital, 40% on rehabilitation and long-term care, informal care and productivity loss explain 10% of total cost for the stroke disease. The results of this study can be used as further development as well as for improvements and investments in health care as well as for development of econometric methods.

## Competing interests

The study was performed upon request from the county council of Västra Götaland and the authors acknowledge financial support from the county council of Västra Götaland. However, none of the authors has received reimbursement, fees, funding, or salary from any other organisation. None of the authors hold any stock or shares in an organisation that may in any way gain or lose financially from the publication of this manuscript, either now or in the future. None of the authors hold or are currently applying for any patents relating to the content of the manuscript or received reimbursements, fees, funding, or salary from an organization that holds or has applied for patents relating to the content of the manuscript. None of the authors have any other financial or non-financial competing interests.

## Authors’ contributions

JP has written the manuscript and has made substantial contributions to analysis. JFN have made substantial contributions to analysis and revising the manuscript. IK have been involved in designing the study, drafting and revising the manuscript. All authors read and approved the final manuscript.

## Pre-publication history

The pre-publication history for this paper can be accessed here:

http://www.biomedcentral.com/1472-6963/12/341/prepub
